# Atezolizumab and stereotactic body radiotherapy in patients with advanced non‐small cell lung cancer: safety, clinical activity and ctDNA responses—the ComIT‐1 trial

**DOI:** 10.1002/1878-0261.13330

**Published:** 2022-11-22

**Authors:** Henrik Horndalsveen, Tine Norman Alver, Astrid Marie Dalsgaard, Lotte Victoria Rogg, Nina Helbekkmo, Bjørn Henning Grønberg, Tarje Onsøien Halvorsen, Christina Ramberg, Vilde Drageset Haakensen, Åsa Kristina Öjlert, Maria Moksnes Bjaanæs, Åslaug Helland

**Affiliations:** ^1^ Department of Oncology Oslo University Hospital Norway; ^2^ Department of Cancer Genetics, Institute for Cancer Research Oslo University Hospital Norway; ^3^ Department of Clinical Medicine University of Oslo Norway; ^4^ Department of Pulmonology University Hospital of North Norway Tromsø Norway; ^5^ Department of Clinical and Molecular Medicine NTNU, Norwegian University of Science and Technology Trondheim Norway; ^6^ Department of Oncology, St. Olavs Hospital Trondheim University Hospital Norway; ^7^ Department of Medical Physics Oslo University Hospital Norway

**Keywords:** circulating tumour DNA, immunotherapy, liquid biopsy, NSCLC, radiotherapy

## Abstract

The introduction of immune checkpoint inhibitors has transformed the treatment landscape of metastatic non‐small cell lung cancer. However, challenges remain to increase the fraction of patients achieving durable clinical responses to these drugs and to help monitor the treatment effect. In this phase II trial, we investigated the toxicity, systemic responses and circulating tumour DNA responses in patients (*n* = 21) with advanced non‐small‐cell lung cancer treated with atezolizumab and stereotactic body radiotherapy in the second or later line. We found the combined treatment to be safe with grade 3 toxicity reported in three patients. As the best overall response, four patients had a partial response, eight had stable disease and five had progressive disease. Median overall survival time was still not reached after a median follow‐up of 26.5 months and 10/15 patients with programmed death‐ligand 1 negative tumours were alive >18 months after the start of the study treatment. ctDNA was detectable at baseline in 11 patients. A rapid decline in ctDNA to <30% of baseline levels was seen in three patients, two of which were radiographic responders and one was considered clinically benefiting from therapy for almost 1 year.

## Graphical abstract

Immunotherapy has transformed the treatment landscape of advanced non‐small cell lung cancer. However, challenges remain to increase the fraction of patients achieving durable responses to these drugs and to help monitor the treatment effect. In this clinical trial, we investigated the tolerability, efficacy and circulating tumour DNA responses in patients with metastatic lung cancer treated with atezolizumab combined with radiotherapy.

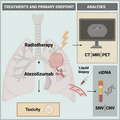

**Abbreviations**
AF: allele frequency
ALK: anaplastic lymphoma kinase
BED: biologically effective dose
cfDNA: cell‐free DNA
cfNA: cell‐free nucleic acid
CNV: copy number variants
CR: complete response
CTCAE: common terminology criteria for adverse events
ctDNA: circulating tumour DNA
EDTA: ethylenediaminetetraacetic acid
EGFR: epidermal growth factor receptor
ERBB2: erythroblastic oncogene B
FFPE: formalin‐fixed paraffin‐embedded
GTV: gross tumour volume
ICI: immune checkpoint inhibitor
indels: insertion–deletion mutations
KRAS: Kirsten rat sarcoma viral oncogene homolog
LOD: limit of detection
MET: mesenchymal–epithelial transition factor
NE: not evaluable
ng: nanogram
NGS: next‐generation sequencing
NSCLC: non‐small‐cell lung cancer
ORR: objective response rate
OS: overall survival
PD‐1: programmed cell death protein‐1
PD‐L1: programmed death‐ligand 1
PFS: progression‐free survival
PR: partial response
PS: performance status
PTV: planning target volume
RT: radiotherapy
SBRT: stereotactic body radiotherapy
SD: stable disease
SNV: single nucleotide variants

## 1 Introduction

Lung cancer is one of the main contributors to the global cancer burden with an estimated 2.21 million new cases and 1.79 million deaths worldwide in 2020 [1]. During the last years, the median overall survival (OS) among patients with advanced non‐small cell lung cancer (NSCLC) has increased, partly due to the development and emerging use of immune checkpoint inhibitors (ICIs) targeting programmed death 1 (PD‐1) and PD‐ligand 1 (PD‐L1) [2, 3]. Unfortunately, durable clinical responses to these drugs in monotherapy are limited to a subset of NSCLC patients with approximately 15–20% of unselected pretreated patients achieving an objective response [4, 5, 6]. One promising approach to increasing response rates to PD‐1/PD‐L1 inhibitors is combining them with radiotherapy [7]. Radiotherapy is widely known to induce tumour cell death through DNA damage. Interestingly, irradiation has also been shown to have an immunomodulating effect. By causing immunogenic cell death, radiotherapy can induce tumour antigen presentation to stimulate the immune system and thus convert the tumour into an *in situ* vaccine [8]. Further, irradiation can increase the expression of PD‐L1, stimulate the release of pro‐inflammatory cytokines and reduce myeloid‐derived suppressor cells, making the tumour microenvironment more attackable to PD‐1/PD‐L1 inhibitors [9, 10, 11]. All of this might contribute to an abscopal effect where localized radiotherapy can initiate an antitumour response distant from the primary target [12]. Several studies have found the combination of radiotherapy and PD‐1/PD‐L1 inhibitors to be well tolerated, though further trials are needed to explore the clinical benefit of this therapeutic approach [7, 13, 14, 15, 16].

Assessment of therapeutic efficacy in patients with NSCLC ismainly based on radiographic scans. This evaluation method has its limitations when monitoring immunotherapy response. Checkpoint inhibitors sometimes cause a transient increase in tumour size due to infiltration of immune cells, pseudoprogression and response to ICIs on CT scans can be delayed [17, 18, 19]. Consequently, there is a need for new biomarkers to better select patients who will benefit from immunotherapy and to help monitor the effect of treatment in the early phase.

Cell‐free DNA (cfDNA) are degraded fragments of cellular DNA, usually 140–200 base pairs in length, found in the circulation of both healthy and diseased individuals [20]. In cancer patients, a fraction of cfDNA constitutes circulating tumour DNA (ctDNA) released from tumour cells to the bloodstream through apoptosis, necrosis or active secretion [21, 22]. ctDNA has a short half‐life ranging between 15 min and a few hours, and the level tends to be higher in patients with metastatic disease. Analysis of ctDNA obtained by liquid biopsies has several tempting aspects: it is minimally invasive and can easily be repeated during therapy, it might reflect intra‐ and intertumoural heterogeneity in patients and enables sustained tracking of genetic alterations over the course of treatment [23, 24, 25]. Evolving data indicates that changes in ctDNA can be used to estimate clinical outcomes in patients receiving anti‐tumour treatment [26]. Several studies have found that there is a correlation between early reduction in ctDNA‐levels and therapeutic efficacy in NSCLC patients treated with anti‐PD‐1/PD‐L1‐therapy [17, 25, 27, 28, 29, 30, 31, 32].

In this study, we investigated the combination of a PD‐L1 inhibitor, atezolizumab and radiotherapy in chemotherapy‐pretreated patients with advanced NSCLC. We here present the clinical outcomes, including therapeutic responses and adverse events, as well as mutations detected in ctDNA, changes in ctDNA during treatment and how they relate to therapy responses at an individual level.

## 2 Materials and methods

### 2.1 Patients

The Combinatory ImmunoTherapy‐1 (ComIT‐1) trial is a multi‐center phase II trial conducted at three university hospitals in Norway. In this study, we included patients with advanced NSCLC (stages III–IV) previously treated with a platinum doublet. Eligible patients were > 18 years of age with an ECOG performance status score of 0 or 1, adequate organ and hematologic functions, measurable disease according to Response Evaluation Criteria in Solid Tumours version 1.1 (recist 1.1) and a tumour lesion suitable for stereotactic body radiotherapy. Key exclusion criteria were significant cardiac, pulmonary or other medical illness that would limit activity or survival, previous treatment with a PD‐1/PD‐L1 inhibitor, a history of drug‐induced or idiopathic pneumonitis, active/untreated brain metastases and medical conditions requiring > 30 mg·day^−1^ of prednisone or equivalent. Patients with *EGFR* mutation or *ALK* translocation were eligible if they had previously been treated with a tyrosine kinase inhibitor.

### 2.2 Study design and treatment

Patients received the PD‐L1 inhibitor atezolizumab concomitant with SBRT. Atezolizumab was administered at a fixed dose of 1200 mg intravenously every 3 weeks for a maximum of 2 years until intolerable toxicity or no clinical benefit as judged by the investigator. SBRT 6 Gy × 3, fractions 1 day apart, was given towards one or two tumour lesions, minimum 2 cm^3^ in volume as determined by gross tumour volume (GTV), between the first and second infusion of atezolizumab. Metastatic lesions were preferred for irradiation, and at least one tumour lesion should not be irradiated to act as a target lesion for RECIST evaluation. The primary endpoint was toxicity. Secondary endpoints included response rates, progression‐free survival (PFS), duration of response, OS, abscopal effects and quality of life. Exploratory endpoints included immunological response, tumour evolution, dynamics in the tumour microenvironment and biomarkers of clinical response. The study was conducted in accordance with the ICH E6 guidelines for Good Clinical Practice, the principles of the Declaration of Helsinki [33] and was approved by the regional committee for medical and health research ethics. Informed written consent was obtained from all patients. The trial is registered on www.clinicaltrials.gov (ClinicalTrials.gov identifier: NCT03644823).

### 2.3 Clinical outcomes

Tumour evaluation with CT scans was performed at baseline and then every 9 weeks for the first 6 months, thereafter every 12 weeks. Patients underwent supplemental MRI and 18F‐FDG‐PET‐scans if clinically indicated. Tumour response was assessed according to recist 1.1 [34], including only lesions outside the radiation field. To calculate the radiographic tumour burden we used the sum of the diameters (longest for non‐nodal lesions and short axis for nodal lesions) for all target tumour lesions. Patients achieving a complete response (CR) or partial response (PR) were considered radiographic responders. PFS was defined as the time from initiation of treatment to progression or death from any cause. OS was defined as the time from treatment initiation to death. OS and PFS were estimated using the Kaplan–Meier method and follow‐up time was calculated using reverse Kaplan–Meier. Adverse events were registered and graded according to the National Cancer Institute Common Terminology Criteria for Adverse Events (ctcae) version 4.0 (U.S. Department of Health and Human Services, National Institutes of Health, National Cancer Institute, Bethesda, MD, USA).

### 2.4 ctDNA analysis

Blood samples were collected at baseline, during radiotherapy, at every evaluation time point and at disease progression. Three 10 mL‐EDTA tubes of peripheral blood were taken, and plasma was separated by centrifugation at 1000 ***g*** for 10 min within 1 h of collection, immediately aliquoted and conserved at −80 °C. Total cfNA were isolated from 2 mL EDTA‐plasma using MagMAX Cell‐Free Total Nucleic Acid Isolation Kit (Thermo Fisher Scientific, Waltham, MA, USA), according to the manufacturer's recommendations. The elution volume was 20 𝜇L. Targeted Sequencing Libraries were prepared manually using the Oncomine™ Pan‐Cancer Cell‐Free Assay. Inputs were 1.46–77.38 ng cfNA (median 7.69 ng). Libraries were quantified with qPCR, amplified and loaded on Ion 540 chips by Ion Chef and sequenced on the Ion GeneStudio™ S5 system (Thermo Fisher Scientific). Output data were analyzed using the ion reporter software (Thermo Fisher Scientific). We considered all single nucleotide variants (SNVs) and indels with an allele frequency (AF) ≥ 0.1 at baseline as significant provided that the AF surpassed the limit of detection (LOD) calculated using the given DNA input. Copy number variants (CNVs) had to exceed 1.4× fold change to be reported. In case of multiple mutations, we used the one with the highest AF before the start of treatment for longitudinal monitoring.

To confirm that genetic alterations detected in blood originated from tumour tissue, we did a genomic analysis of formalin‐fixed paraffin‐embedded (FFPE) tissue samples collected before the start of study treatment or at the time of diagnosis if no dedicated pre‐treatment biopsy was available. Targeted libraries were prepared on the Ion Chef from 20 ng of tumour DNA with the Ion AmpliSeq™Cancer Hotspot Panel v2, quantified with qPCR, amplified and loaded on Ion 540 chips by Ion Chef and sequenced on the Ion GeneStudio™ S5 system (Thermo Fisher Scientific). Output data were analyzed using the Ion Reporter software (Thermo Fisher Scientific).

## 3 Results

### 3.1 Patient characteristics

We enrolled 21 patients; 13 men and 8 women, with a mean age of 61.7 years, in the trial between September 2018 and February 2020. (Table 1). All patients had stage IV disease at enrollment. Twenty patients were in performance status (PS) 0–1. Histologically, 16 cases were classified as adenocarcinoma of which two had sensitizing *EGFR* mutations with progression on EGFR inhibitors and platinum‐based chemotherapy. Fifteen patients were PD‐L1 negative and 13 received atezolizumab as second‐line treatment.

**Table 1:** Patient characteristics

Sex (*n* = 21)	
Male	13
Female	8
Age: mean (range)	61.7 (50–79)
Smoking	
Current	5
Former	14
Never	0
Unknown	2
Performance status	
0	7
1	13
2	1
≥ 3	0
Histology	
Adenocarcinoma	16
Squamous	1
Large cell neuroendocrine	1
NSCLC not otherwise specified	2
Adenoid cystic	1
Genotype	
EGFR	2
ALK	0
PD‐L1 expression in tissue	
0%	15
1–49%	5
≥ 50%	1
Number of metastatic lesions: median (range)	10 (1–30)
Number of previous lines of systemic treatment	
1	13
2	5
≥3	3

Lung tumours were selected for SBRT in 16 cases. Only one patient received SBRT for more than one lesion. Treatment details regarding radiotherapy can be found in Table 2.

**Table 2:** Radiotherapy details. GTV, gross tumour volume; PTV, planning target volume; RT, radiotherapy.

Patient	Location	GTV (ccm)	PTV (ccm)	PTV dose (Gy); avg (range)	Additional RT during trial
1	Adrenal gland	15.94	101.55	22.4 (15.6–27.0)	
2	Lung	79.70	289.81	22.3 (15.6–27.0)	
3	Lung	7.17	51.66	23.2 (13.6–26.2)	
4	Lung	19.80	101.92	23.3 (14–27)	
5	Lung	6.45	53.92	23.1 (15.4–27)	
6	Lung	13.15	77.16	23.8 (14.4–27)	
7	Lung	5.57	48.37	24.5 (16.7–27)	
8	Lung	26.0	121.33	23.6 (17–27.4)	
9	Lung	44.8	174.75	25.0 (15.3–28.1)	
10	Adrenal gland	14	37.70	23.9 (16.8–27.3)	4 Gy × 5 adrenal gland, 3 Gy × 7 adrenal gland (after progression)
11	Lung	21.1	91.83	23.5 (17.2–27)	
12	Lung	187.6	465.20	24.3 (14.9–27.3)	
13	Liver	14.9	40.76	24.1 (17.4–27.2)	
14^a^	Brain	0.21; 0.31	0.85; 1.20	20.9 (16.9–22.7); 23.1 (15.0–25.4)	3 Gy × 10 femur, 4 Gy × 5 thigh, 4 Gy × 5 pelvis
15	Lung	1.63	29.13	22.9 (17.5–27)	
16	Lung	4.3	37.98	23 (17–27)	
17	Lung	11.32	66.22	22.4 (17.8–26.6)	
18	Lung	17.03	86.3	22.6 (17.7–25.9)	
19	Thoracic wall	28.1	117.54	22.8 (17.7–18.6)	
20	Lung	4.32	35.48	21.9 (17.7–18.5)	
21	Lung	103.7		18.8 (17.1–20.3)	

^a^ To prevent radiation‐induced edema, patient 14 received corticosteroids, i.e., 48 mg of methylprednisolone from the first day of SBRT until 1 day after the last treatment. Steroids were then tapered over 2 weeks.

### 3.2 Toxicity

Fifteen patients (71%) experienced adverse events of any grade possibly or definitely related to treatment. Grade 3 treatment‐related toxicity was reported in three patients; one colitis, one pneumonitis and one skin toxicity. The patient with colitis had to permanently stop treatment due to a recurrent grade 3 event. For the patients with grade 3 pneumonitis and grade 3 skin toxicity, oral prednisolone was administered and atezolizumab was temporarily held for 8 and 3 weeks respectively, until symptoms had recovered to grade 1. In both cases, atezolizumab was successfully restarted. There were no grade 4 or 5 events. The most common adverse events were skin rash (*n* = 6), flu‐like symptoms (*n* = 5) and elevated liver enzymes (*n* = 5, of which four were CTCAE grade 1). Of special interest, pneumonitis occurred in three patients, of which two had received SBRT to lung lesions. In both these patients the pneumonitis was grade 1. Except for these cases, adverse events were mainly systemic in nature and not confined to irradiated areas. A summary of adverse events and relevant treatment‐related toxic effects are shown in Tables 3 and 4, respectively.

**Table 3:** Summary of adverse events.

	Number of patients (%)
All AEs	16 (76%)
Treatment‐related AEs	15 (71%)
Grade 3 AEs	6 (29%)
Treatment‐related grade 3 AEs	3 (14%)
AEs leading to treatment delay or interruption	3 (14%)
AEs leading to discontinuation of treatment	1 (5%)
Treatment‐related deaths	0

**Table 4:** Treatment‐related adverse events. AE, adverse events; ALT, alanine transaminase; AST, aspartate transaminase.

Event	Any grade	Grade 3
Flu‐like symptoms	5 (24%)	0
Fatigue	3 (14%)	0
Diarrhea	4 (19%)	0
Nausea	1 (5%)	0
Dyspnea	3 (14%)	0
Skin rash	6 (29%)	1 (5%)
Hyperthyroidism	4 (19%)	0
Hypothyroidism	2 (10%)	0
Pneumonitis	3 (14%)	1 (5%)
Colitis	3 (14%)	1 (5%)
Pancreatitis	1 (5%)	0
ALT and/or AST elevation	5 (24%)	0
Lipase elevation	4 (19%)	0
Creatinine elevation	1 (5%)	0

### 3.3 Treatment response and survival

**Fig. 1:**
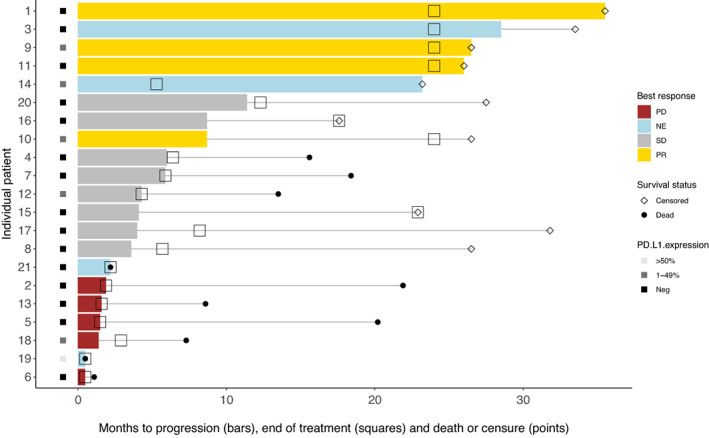
Clinical outcomes including best overall response (colour of the bar), months to progression (length of the bar), months on treatment (square) and months to death or censure (point) for each individual patient. NE, not evaluable; PD, progressive disease; PR, partial response; SD, stable disease.

At the cut‐off date for analysis (6 September 2021), the median follow‐up time was 26.5 months (range 17.6–35.5). Two patients were still receiving treatment. A median of nine doses (range: 1–35) of atezolizumab was administered and five patients completed 2 years of treatment. Median PFS was 4.3 months (95% CI 2.2–8.7). Median OS time was still not reached at the data cut‐off. As the best overall response, four patients had a PR, eight had stable disease (SD) and five had progressive disease (PD). Among the four patients achieving av PR, the median duration of response was 17.8 months. Four patients were not evaluable (NE) according to recist v1.1. Two of these patients (patients 19 and 21) had a rapid symptomatic deterioration probably due to disease progression though not radiographically confirmed. The other two (patients 3 and 14) were considered to have a prolonged (>18 months) clinical utility from treatment without the progression of non‐target lesions or the appearance of new lesions. However, they were not evaluable after radiotherapy, patient 3 due to pneumonitis making the assessment of the target lesion impossible, and patient 14 because both measurable lesions in the hip and pelvis were irradiated shortly after the first infusion of atezolizumab. In general, there was a trend towards two distinctive patterns of response among patients, either a long‐lasting clinical benefit or a rapid worsening of the disease. Eight patients were on treatment for more than 12 months, and three of them were treated beyond radiological progression. In contrast, seven patients had a time to treatment discontinuation < 3 months. Clinical outcomes for the individual patients are shown in Fig. 1.

The efficacy endpoints focused on the systemic responses, and irradiated lesions were excluded as target lesions. However, when reviewing the treatment effect on irradiated lesions, there were no patients whose non‐irradiated lesions responded better than the irradiated ones. All four patients with PR as the best overall response also had a significant reduction of the irradiated tumours, in two cases leading to a complete radiologic disappearance of the tumour.

### 3.4 Treatment response and survival in patients with PD‐L1 negative tumours

The 15 participating patients with PD‐L1 negative tumours had a median of 10 infusions of atezolizumab. As the best overall response, two patients achieved a partial response and seven had SD, creating an objective response rate (ORR) of 13% and a disease control rate of 60%. Median PFS was 4.1 months. Six PD‐L1 negative patients were on treatment and considered clinically benefitting from therapy for more than 1 year, although three of them were treated beyond radiologic progression. Median OS time was still not reached at the data cut‐off, but 10 out of the 15 patients were alive more than 18 months after the start of the study treatment.

### 3.5 ctDNA before treatment

**Fig. 2:**
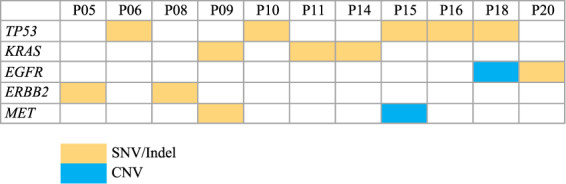
Somatic mutations detected in plasma at baseline. CNV, copy number variants; SNV, single nucleotide variants.

Baseline samples for ctDNA analysis were available from 19 patients. Lung cancer‐associated somatic mutations were identified in 11 of these (Fig. 2). The gene most commonly affected was *TP53* (five patients) followed by *KRAS* (three patients). Three patients had more than one detectable variant. No fusions were detected. From 8 of the 11 patients with cancer‐associated genetic alterations in plasma at baseline, we had FFPE tissue samples available for genomic analysis. In seven of these cases identical mutations were also found in tumour tissue. One patient had a *TP53* mutation and a *MET* amplification was detected in blood, but not in tumour tissue.

### 3.6 Clinical outcomes among patients with oncogenic driver alterations detected in ctDNA at baseline

Six patients had targetable driver mutations other than *KRAS* detected in blood at baseline (two *EGFR*, two *ERBB2* and two *MET*). These patients had in general poor response to atezolizumab with only one patient achieving a partial response and four out of six patients experiencing a rapid progression. In contrast, of the three patients with KRAS mutations two were considered partial responders while the third had a prolonged clinical benefit although not evaluable according to recist v1.1.

### 3.7 ctDNA responses and radiographic responses

**Fig. 3:**
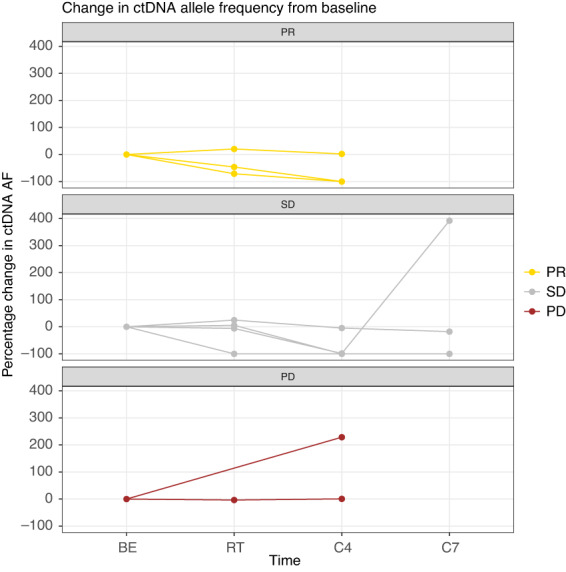
Percentage change in ctDNA level from baseline during the first months of treatment for each patient (*n* = 9), grouped according to the best overall radiographic response. AF, allele frequency; BE, before start of treatment; C4, before atezolizumab infusion number four (day 63); C7, before atezolizumab infusion number seven (day 126); ctDNA, circulating tumour DNA; PD, progressive disease; PR, partial response; RT, during radiotherapy (days 7–14); SD, stable disease.

We examined the relationship between ctDNA responses and radiographic responses for each patient during treatment. ctDNA was quantified using the allele frequencies of mutant tumour‐derived DNA. Of the 11 patients with detectable ctDNA at baseline, one did not have additional blood samples suitable for ctDNA analysis and one did not have measurable disease according to recist v1.1. Figure 3 illustrates the percentage change in AFs during the first months of treatment for the remaining nine patients; three of them were radiographic responders and six of them were non‐responders.

In the responder group, two out of three patients had a rapid decline in ctDNA to <30% of baseline levels within 2 weeks. In both patients, the ctDNA continued to decrease until undetectable levels at the second blood draw 9 weeks after the start of treatment. The third radiographic responder (patient 09) had a slight increase in ctDNA during radiotherapy and did not experience a significant drop in ctDNA. Patients without a radiographic response showed a more variable ctDNA trend. Only one of these six patients had a drop in ctDNA level to < 30% of baseline at 2 weeks. Four patients in this group had minor changes in ctDNA levels at the first checkpoint (< 30% change from baseline). One patient did not have an additional plasma sample before week 9, when the ctDNA had increased dramatically.

When following the sequential changes in ctDNA and radiographic tumour burden for each patient during treatment (Fig. S1), there was a relatively good agreement between ctDNA responses and radiological responses in six of the patients (patients 05, 08, 10, 11, 16 and 18). For the remaining three individuals (patients 09, 15 and 20), the changes in ctDNA did not reflect changes in radiographic tumour burden.

Patient 09 was registered as a responder on CT scans after 11 months. This patient had two detectable cancer‐associated mutations in plasma at baseline, and while the level of the one with the highest AF (*MET*) was quite stable, the second (*KRAS*) reached undetectable levels during treatment. In patient 15, the pre‐treatment alterations in plasma affecting the *TP53*‐ and *MET*‐genes were detected at similar quantities during radiotherapy but were not detectable at later timepoints when the tumour progressed. A potential explanation for this mismatch could be the development of treatment‐resistant clones harbouring other genetic alterations [21]. Patient 20 was observed to ha a rapid decline in ctDNA to undetectable levels and no additional mutations emerged in plasma during treatment. While this patient did not meet the criteria for PR, he had a prolonged SD on scans and was considered clinically benefiting from therapy for almost 1 year. Furthermore, the AF at baseline was very low, and we cannot rule out a false positive test.

## 4 Discussion

The ComIT‐1 trial investigated primarily the toxicity, secondarily the systemic responses and exploratively the ctDNA responses in metastatic NSCLC patients treated with atezolizumab and stereotactic body radiotherapy. In accordance with previous studies [7, 13, 14, 15, 16], we found the combined treatment to be well tolerated yielding similar rates of toxicity as reported with atezolizumab monotherapy [4, 5]. Luke et al. [14] observed in their prospective trial of multisite SBRT followed by pembrolizumab that toxicity most frequently appeared in the anatomic region that was irradiated. A similar pattern was not seen in our study where patients mainly experienced systemic side effects. In particular, the relative occurrence of pneumonitis and gastrointestinal toxicity was not higher in patients receiving radiotherapy for lung lesions and abdominal lesions, respectively. This discrepancy might reflect the differences in radiation load given to patients in the two trials, where Luke et al irradiated two to four lesions with a higher dose (SBRT of 30–50 Gy) than in our study. When the target volume is larger and the radiation dose is higher, the local toxic effects are expected to be more pronounced.

Due to a small sample size, it is not possible to draw solid conclusions from our study, and we have chosen a descriptive approach. The objective response rate in our trial was similar to those reported with PD‐1/PD‐L1 inhibitors as monotherapy for pretreated patients with advanced NSCLC [4, 5, 6]. It remains unclear whether adding radiotherapy to ICIs increases systemic response rates and survival in stage IV NSCLC [7, 35]. The PEMBRO‐RT trial compared SBRT and pembrolizumab prospectively to pembrolizumab alone, with a higher 12‐week ORR (36% vs 18%) and longer median PFS (6.6 vs 1.9 months) found in the combined treatment arm [15]. In contrast, a study conducted by Welsh et al. [16] randomizing patients with metastatic NSCLC to receive pembrolizumab with or without radiotherapy did not show significant differences in ORRs or PFS between the two groups. Major distinctions between these studies were the patient populations and the radiotherapy regimens. Interestingly, subgroup analysis from both trials indicates that adding radiotherapy may be more advantageous for patients without a high PD‐L1 expression [15, 16].

In our study, the majority of participating patients had PD‐L1 negative tumours, and a substantial number of these experienced a durable clinical utility. Out of the 15 PD‐L1 negative patients, 6 (40%) underwent treatment with atezolizumab for more than 12 months and 10 (67%) were alive more than 18 months after the start of the study treatment. These numbers are encouraging, considering subgroup analysis from the phase III OAK trial which showed that atezolizumab was superior to docetaxel in previously treated NSCLC patients [4]. In that trial, PD‐L1 negative patients had an ORR of 8% and a median OS of 12.6 months.

Nevertheless, a significant proportion of our patients did not respond to the study treatment. One reason might be that the SBRT schedule was not ideal. The optimal radiation dose, fractionation and timing to generate an immune response and enhance the effect of combining radiotherapy with immunotherapy remain to be defined. Preclinical models suggest that prior radiotherapy upregulates PD‐L1 expression and thus facilitates later administration of ICIs [11]. In a meta‐analysis performed by Geng et al. [35], subgroup analysis showed that the combined treatment was more effective when radiotherapy was given before checkpoint inhibitors. In contrast, Dagoglu et al. [12] found that most reported cases of abscopal response occurred when immunotherapy was administrated prior to or concurrent with radiotherapy.

In our trial, radiotherapy was given as SBRT, but with significantly lower doses than standard curative stereotactic radiotherapy. This radiotherapy dosing was chosen to induce immunogenic cell death while being well tolerated and was in accordance with other studies testing the combination of immunotherapy and radiation [36]. Increasing evidence suggests SBRT to be more favorable to combine with ICIs than conventional radiotherapy considering out‐of‐field responses and survival benefits [11, 16, 35]. The immunologic effect of radiation seems to be dose‐dependent and the optimal dose might be higher than 6 Gy × 3 selected in our study [11]. In a meta‐analysis, Marconi et al. found the occurrence rate of an abscopal effect in preclinical models to be directly correlated with the biologically effective dose (BED), with a BED of 60 Gy necessary to generate an abscopal effect in 50% of cases [37]. However, to what extent this also applies in clinical practice and with respect to the synergistic effect of radiotherapy and immunotherapy is unknown.

Multisite SBRT represents another interesting approach to optimize the combined treatment of radiotherapy and immune checkpoint inhibitors [14]. SBRT directed towards multiple tumour lesions would improve local disease control and reduce overall tumour burden to give PD‐1/PD‐L1 inhibitors time to exert their effect. In addition, multisite irradiation is more likely to activate a potent and heterogeneous systemic antitumour immunity [11]. More trials are warranted to confirm this treatment to be well tolerated and to investigate the clinical benefit of the comprehensive approach.

In advanced NSCLC, there is a need for new predictive biomarkers to better identify patients who will benefit from immune checkpoint blockade and to help monitor the treatment effect. A promising tool in this setting is ctDNA. Previous studies have demonstrated a strong correlation between longitudinal ctDNA changes, radiographic responses and clinical outcomes in stage IV NSCLC patients treated with ICIs [17, 21, 25, 27, 28, 29, 30]. Most patients in our trial had concordant ctDNA and radiographic responses. In particular, a rapid decline in ctDNA to < 30% of baseline levels within 2 weeks was found only among patients responding to CT scans or deriving a long‐lasting clinical benefit from treatment. However, in three patients (patients 09, 15 and 20), the changes in AF of mutant tumour‐derived DNA did not match the changes in radiographic tumour burden. One open question is whether these aberrations are solely due to methodological concerns or attributed to tumour biology characteristics. It must be emphasized that the calculation of radiographic tumour burden was only based on the sum of diameters for target lesions, which may introduce bias in the results for polymetastatic patients. The correspondence between ctDNA and radiographic responses might have looked different if we had a complete volume estimate for all tumour lesions.

A potential advantage of ctDNA is its rapid kinetics. Several studies have reported that the ctDNA response considerably precedes the radiological response in NSCLC patients treated with immunotherapy [17, 25, 27]. In our trial, this was seen in particular in two responders. This early pattern of response can help overcome the challenges of pseudoprogression and delayed response on CT scans seen in some patients during the first months of ICI treatment. Analyzing ctDNA during treatment using next‐generation sequencing (NGS) enables continuous monitoring of tumour evolution and identification of emerging mutations and acquired resistance mechanisms before a subclonal evolution manifests as a progression on images. This may provide clinical guidance concerning when to stop ICIs and the choice of drug in the next line of therapy. It must be emphasized that ctDNA data and imaging data are offering complementary information regarding the medical landscape. While ctDNA analysis can identify genetic alterations and help guide clinical decisions in certain oncological situations as those mentioned above, radiographic scans obviously give a superior disease map necessary to understand the patient's symptoms and initiate local treatment.

Some studies have reported a transient flare‐up of AF within the first 14 days after the start of immunotherapy in a subgroup of patients, possibly due to the increased release of DNA from dying tumour cells [17, 29, 38]. In theory, radiation therapy given during the same period of time could increase the number of tumour cells killed and amplify such a temporary spike. Our observations do not support this hypothesis. In two patients the ctDNA did flare up transiently during radiotherapy, but these flares were minor and not followed by major drops in ctDNA level as we would have expected if they were caused by massive death of cancer cells. The lack of significant spikes of ctDNA during radiotherapy could be due to small volumes of irradiated lesions. Another possible explanation might be the timing of the blood draw relative to the radiotherapy since ctDNA has a very short half‐life and is cleared from the blood within a few hours.

The subtype of mutation detected in blood seemed to impact the response to atezolizumab in our trial. In accordance with previous reports, we found that patients with a targetable driver mutation (*EGFR*, *ERBB2* and *MET*) had a poor treatment response, while patients with a *KRAS* mutation detected in plasma‐derived more benefit. This is well documented in tissue biopsies and the same response pattern is expected to be found with liquid biopsies [4, 28, 39, 40].

A major limitation is the small and heterogeneous study population and the non‐randomized design, which makes it difficult to draw firm conclusions. To capture the role of SBRT in the combined approach with respect to improving PFS and response rates, a larger trial containing a comparison group receiving ICI alone is needed. Furthermore, pre‐treatment ctDNA was detected in only 11 of the 21 enrolled patients and due to incomplete tissue genotyping data, we were not able to confirm the concordance between mutations detected in plasma and mutations in tissue for all patients. In theory, mutations identified in plasma could represent contamination from germline variants and clonal hematopoietic mutations and not be attributed to tumour cells. We could have reduced this risk by doing paired peripheral blood mononuclear cell NGS or by excluding mutations with AF > 20% in all patient samples [41]. In one patient with FFPE tissue samples available for genomic analysis, mutations detected in blood were not recovered in tumour tissue. In this patient, the only available tissue sample was a surgical specimen obtained 2 years before study inclusion, which makes emerging mutations during the course of the disease a likely explanation for this mismatch. Additionally, formalin fixation could cause extensive degradation of nucleic acids making mutant DNA unrecognizable after several years. A final limitation is the low AFs and low amount of DNA input in some of the ctDNA‐analysis. We considered all SNVs and Indels with an AF ≥ 0.1 at baseline as significant provided that the AF surpassed the limit of detection calculated using the actual DNA input. However, this might cause a lack of specificity and we have to approach minor changes in AF during treatment with caution.

## 5 Conclusions

In conclusion, concurrent atezolizumab and SBRT were safely administered in metastatic NSCLC patients, yielding encouraging results in a subset of patients, including those with PD‐L1 negative tumours. Additional trials are needed to evaluate the optimal radiation dose, fractionation and sequence to increase systemic response rates. ctDNA holds potential as a dynamic biomarker in lung cancer patients receiving immunotherapy, however, questions remain concerning the robustness and practical implementation.

## Conflict of interest

Henrik Horndalsveen: Advisory board: Janssen. Honoraria: AstraZeneca, Pfizer, Roche. Bjørn Henning Grønberg: Advisory board: AstraZeneca, BMS, Debiopharm, Eli Lilly, Janssen, MSD, Novartis, Pfizer, Roche, Takeda. Honoraria: AstraZeneca, Bayer, BMS, Bohringer Ingelheim, Debiopharm, Eli Lilly, MSD, Novartis, Pfizer, Pierre Fabre, Roche, Sanofi, Takeda. Research funding: AstraZeneca, Roche. Spouse: Employee and shareholder of Eli Lilly and Company. Tarje Onsøien Halvorsen: Honoraria: AstraZeneca, MSD, Pierre Fabre, Pfizer. Travel support: AstraZeneca, MSD. Vilde Drageset Haakensen: Advisory boards: Novartis, Astra Zeneca, Pfizer. Honoraria: BMS, Astra Zeneca, Takeda. Åslaug Helland: Financial support and/or study drug from AstraZeneca, Roche, Novartis, Incyte, Eli Lilly, Ultimovacs and BMS, in association with clinical studies. Adv board: AstraZeneca, BMS, Janssen, MSD, Pfizer, Roche, Takeda, Sanofi, Bayer, Abbvie. The remaining authors declare that they have no competing interests.

## Author contributions

HH: Clinical investigator, analysis of data, drafting the manuscript. TNA: Plasma and tissue sequencing, analysis of data. AMD: Plasma and tissue sequencing, analysis of data. LVR: Clinical investigator. CR: Analysis of data. NH: Clinical investigator. BHG: Clinical investigator. TOH: Clinical investigator. VDH: Clinical investigator. ÅKÖ: Analysis and visualization of data. MMB: Clinical investigator. ÅH: Principal investigator, attract funding, writing protocol, analysis of data. All authors revised and approved the final version of the manuscript.

### Peer review

The peer review history for this article is available at https://publons.com/publon/10.1002/1878‐0261.13330.

## Supporting information


**Fig. S1.** Plasma levels of ctDNA and overall radiographic tumour burden during treatment for each patient.

## Data Availability

The data that supports the findings of this study are available from the corresponding author upon reasonable request.
