# Supplementary material for: Atezolizumab and stereotactic body radiotherapy in patients with advanced non‐small cell lung cancer: safety, clinical activity and ctDNA responses—the ComIT‐1 trial
Source: Mol Oncol. 2022 Nov 22;17(3):487–98. doi: 10.1002/1878-0261.13330 (PMC9980306; doi:10.1002/1878-0261.13330)
Supplement: Supplementary file 1 — Fig. S1. Plasma levels of ctDNA and overall radiographic tumour burden during treatment for each patient. [file MOL2-17-487-s001.pdf]

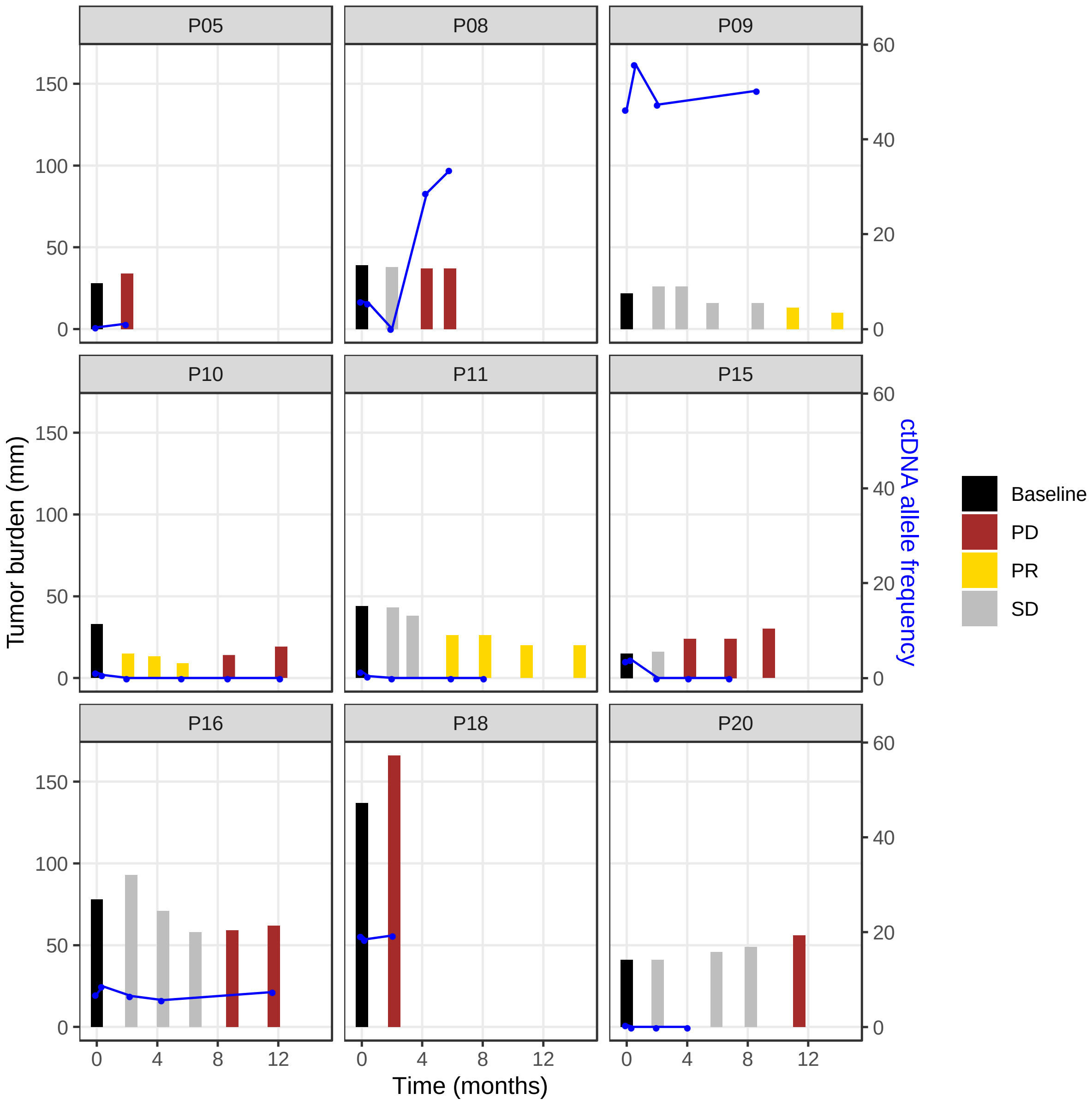

Figure S1 Plasma levels of ctDNA and overall radiographic tumor burden during treatment for each patient. The ctDNA allele frequencies are presented as blue lines. Tumor burden quantified as the sum of the diameters of all target lesions are presented as columns in different colors depending on the response. ctDNA, circulating tumor DNA; PD, progressive disease; PR, partial response; SD, stable disease.
